# Multidrug-resistant ESBL *E. coli* in urban surface waters and public health implications: A Case Study from Goranchatbari, Dhaka

**DOI:** 10.1016/j.heliyon.2025.e42219

**Published:** 2025-01-23

**Authors:** Md. Sakib Hossain, Ahmed Ishtiaque Amin Chowdhury, Mohammad Rafiqul Islam, Ripan Kirtunia, Md. Foysal Abedin, Mohammad Atique Ul Alam, Shampa, Sonia Binte Murshed, Md Shadman Sakib, Siam Alam, M. Shahjahan Mondal, Zahid Hayat Mahmud

**Affiliations:** a Laboratory of Environmental Health, Health Systems and Population Studies Division, International Centre for Diarrhoeal Disease Research, Bangladesh (icddr,b), Dhaka, 1212, Bangladesh; b Institute of Water and Flood Management (IWFM), Bangladesh University of Engineering and Technology (BUET), Dhaka, 1000, Bangladesh; c Department of Civil and Environmental Engineering, Virginia Tech, Blacksburg, VA, 24061, USA

**Keywords:** Surface water, Waterborne diseases, ESBL *E. coli*, Multidrug resistant, Pathogenicity, Public health, Bangladesh

## Abstract

Surface water pollution from rapid urbanization, industrialization, inadequate sanitation, and excessive agrochemical use is a global crisis. In developing countries, water quality is one of the most urgent environmental issues. The presence of pathogenic extended spectrum β-lactamase (ESBL) producing *E. coli* in surface water poses a critical public health concern by increasing the risk of waterborne diseases and spreading multidrug-resistant (MDR) infections. This study addresses the severity of surface water pollution in urban Bangladesh, focusing on the molecular characterization of ESBL *E. coli* in surface waters from the Goranchatbari sub-catchment of Dhaka. Isolates of ESBL *E. coli* underwent analysis for the major ESBL and pathogenic genes, antibiotic susceptibility, biofilm formation, and genetic diversity. However, the existence of *E. coli* was confirmed in every sample. Among 266 isolates, 62 (23.31 %) were phenotypically positive for ESBL, with 58 (93.55 %) carrying at least one of the four ESBL genes: CTX-M, TEM, SHV, and OXA. CTX-M was the most prevalent, found in 55 (88.71 %) isolates. Regarding pathogenicity, 25 (40.32 %) isolates were enteric pathogens, including 24 ETEC and a single EIEC. Four non-diarrheagenic isolates were extraintestinal pathogenic *E. coli* (ExPEC), capable of causing diseases beyond enteric infections. All ESBL isolates were MDR, with high resistance to β-lactam antibiotics, and capable of forming biofilms at 25 °C and 37 °C. ERIC-PCR analysis grouped the isolates into 14 distinct clusters at a 75 % similarity matrix. These water sources critically threaten public health by contaminating nearby freshwater sources with ESBL-producing pathogenic isolates, leading to various hard-to-treat waterborne diseases and risking aquatic biota by deteriorating water quality. Immediate public awareness, proper water treatment, and precise environmental management are needed before using this water for any purpose.

## Graphical abstract

**Figure:**
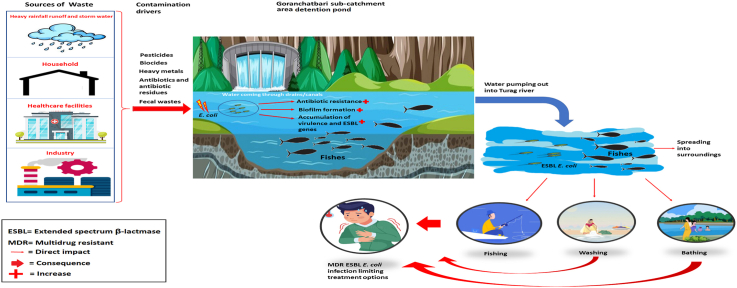

## 1 Introduction

Improper management and disposal of industrial effluents, agricultural runoff and domestic wastes deteriorate surface water quality and threaten the ecosystems and public health around the globe. In developing countries like Bangladesh, the problem is particularly severe [1]. Rapid urbanization and industrialization, combined with poor sanitation infrastructure, result in the frequent dumping of untreated sewage and industrial effluents into rivers, canals and lakes. The lack of effective wastewater treatment facilities further aggravates the situation, making it one of the most urgent environmental challenges. Rivers, which are key sources of surface water, are vital for domestic, agricultural, and industrial uses and play a crucial role in human civilization. Unfortunately, surface water is highly vulnerable to pollution due to the easy access to waste and wastewater [2]. In recent times, rivers in Bangladesh have experienced increased pollution due to rapid population growth, unregulated riverbank development, urban expansion, unplanned industrialization and agricultural activities [1]. The indiscriminate discharge of untreated wastewater and solid waste into the water bodies significantly lowers water quality. Additionally, in areas with inadequate sanitation, direct disposal of human feces into the water bodies greatly contaminates surface water [3].

Escalating pollution levels, especially in terms of biological parameters, have heightened concerns regarding the dissemination of antibiotic resistance in the surroundings. In fact, antibiotic resistance is now recognized as a global public health issue, spreading in the environment through antibiotic resistance genes (ARGs) and antibiotic resistant bacteria (ARB) due to the overuse, and misuse of antibiotics in human, animal, agricultural and aquaculture sectors [4]. Antibiotic resistance develops in bacteria due to the antibiotic residues that might be released into the environment through several routes. The common pathways of antibiotic resistance include hospitals, wastewater, sewage, agrarian activities, foodstuff, and populations of wildlife, while the influencing factors are traces of antibiotics, heavy metals, natural processes, and climate change [5]. Pharmaceutical industries are a major source of antibiotic residues in the environment, particularly in surface water. For instance, a lake in India was found to have approx. 6.5 mg/L of ciprofloxacin resulting from the disposal of wastewater from a cluster of 90 pharmaceutical factories [6]. On the other hand, metronidazole was detected exceeding the safe limit which was about 300 times higher than the threshold in the Kirtankhola river of Bangladesh owing to the industrial operations of nearby pharmaceuticals and the existence of wastewater disposal along the river [7]. Additionally, about 40–90 % of antibiotics consumed by humans for therapeutic and prophylactic purposes are excreted through urine and feces, eventually reaching the environment [4]. Similarly, use of the antibiotics in husbandry, agriculture and aquaculture also contributes to antibiotic residues in the environment. Previous studies have reported the presence of antibiotic residues in aquaculture water as well as in aquaculture products in India, Indonesia, Thailand and Bangladesh [8]. Although residual concentrations in the environment typically range from ng/L to μg/L, and are much lower than administered doses, these concentrations are sufficiently high to provoke antibiotic resistance in environmental microbes [9,10].

Antimicrobial resistance (AMR) in bacteria may result from a variety of different methods, such as enzymatic inactivation of antibiotics, modification of antibiotic targets, and active efflux processes [11]. One potent method of acquired resistance involves enzymatic antibiotic hydrolysis by β-lactamases, particularly Extended Spectrum β-lactamases (ESBLs) [12]. ESBLs are capable of hydrolyzing β-lactam antibiotics, including cephalosporins from the first to the fourth generation, as well as aztreonam, which are sensitive to β-lactamase inhibitors [13]. Of different mechanisms, production of ESBLs is among the most well-known approaches by which Enterobacteriaceae, such as *E. coli* and *Klebsiella pneumoniae*, develop resistance to β-lactam antibiotics [13,14]. Environmental water sources, e.g., rivers, canals and lakes, are susceptible to contamination by *E. coli* originating from humans and other warm-blooded animals. Potential human sources of contamination include wastewater discharge, sewage leaks, malfunctioning septic systems and drain fields. Though *E. coli* is a harmless member of normal gut flora in warm-blooded animals, certain strains of them are capable of causing life-threatening illness [15]. Their pathogenesis can be both intestinal and extra-intestinal. There are currently six well-characterized pathotypes of *E. coli* that cause diarrhea and are capable of infecting people [16]: enteropathogenic *E. coli* (EPEC), enteroinvasive *E. coli* (EIEC), enterohemorrhagic *E. coli* (EHEC), enteroaggregative *E. coli* (EAEC), enterotoxigenic *E. coli* (ETEC), and diffusely adherent *E. coli* (DAEC). The *E. coli* pathotypes capable of causing diseases unrelated to the digestive tract are known as extraintestinal pathogenic *E. coli* (ExPEC) [17], e.g. uropathogenic *E. coli* (UPEC), meningitis-associated *E. coli* (MNEC) etc. Presence of pathogenic *E. coli* in drinking water is associated with high risk of enteric diseases [18].

As the capital of Bangladesh, Dhaka is one of the most rapidly urbanized cities in the world with a population of over 10.2 million living in approximately 300 square kilometers [19]. Due to insufficient sewerage pipelines, much of the untreated sewage is disposed in the internal drains and canals [20]. These drains and canals of Dhaka are increasingly being polluted by the city's numerous industrial units and domestic sewerage lines due to the dumping of large volumes of toxic wastes containing fecal wastes, antibiotic residues, biocides and heavy metals, which might contribute to the rise of antibiotic resistance. Often storm-induced or heavy rainfall runoffs carrying fecal wastes further increase *E. coli* counts in water by several folds [[21], [22], [23]]. Contamination of surface water in Dhaka city by resistant bacteria has been reported in previous studies. Kamruzzaman et al. found the existence of multidrug-resistant (MDR) *E. coli* in the water of two lakes in Dhaka city [24]. Haque et al. detected the existence of the ESBL producing MDR bacteria, e.g. *E. coli*, *K. pneumoniae*, and *Enterobacter cloacae*, in a lake of Dhaka city [25]. Islam et al. found New Delhi Metallo-β-Lactamase-1 producing MDR *K. pneumoniae*, *E. coli*, *Acinetobacter* spp., and *Enterobacter* spp. in surface water of the city [26]. Rabbani et al. detected multidrug and pandrug resistant *E. coli*, and MDR *K. pneumoniae* in disposed untreated hospital wastewater in Bangladesh [27]. The presence of antibiotic resistant bacteria in healthcare facilities and wastewater marks the transmission of antibiotic resistance to the surface water of Bangladesh.

Due to the rapid and unplanned expansion of the city, Dhaka faces severe challenges in maintaining its drainage and sewerage system. Goranchatbari is one of the largest sub-catchments of the city containing a detention pond that receives accumulated stormwaters and wastewater through small drains and canals (locally known as “khal”), from where water is then pumped out to the adjacent Turag river [28]. Therefore, the water quality of the detention pond, influenced by the local drains and canals, impacts the water quality of the Turag river. Previous studies on water quality in Dhaka city have focused on isolated rivers, lakes, beels, canals/khals, and/or ponds but have not covered a complete environmental transmission pathway (disposal into drains, travel through local canals/khals towards larger khals that discharge into detention pond and finally to the river) [[29], [30], [31], [32], [33]]. In addition, a few studies furnish the perception of the surface water contamination with *E. coli* in this city, which is limited [24,25,33]. Therefore, this study focuses on the investigation of the occurrence and features of ESBL *E. coli* in distinct inland surface water bodies within the Goranchatbari sub-catchment of Dhaka city, Bangladesh, aiming to highlight the public health implications of such contamination in an urban setting. The characterization of these ESBL *E. coli* isolates included their ESBL gene profiles, pathogenicity, antibiotic resistance patterns, biofilm formation capacity and genetic variations over an entire year.

## 2 Results

### 2.1 ESBL *E. coli* prevalence in environmental samples and their seasonality

All 28 samples collected from 7 locations in the Goranchatbari sub-catchment over 4 rounds were contaminated with varying levels of *E. coli* (Fig. 2). From the Fig., it's apparent that counts are slightly higher in the wet season by 1.62 % compared to the other seasons. On average, in the wet season, each sample contained 8.80 $\times 10^{6}$ CFU/100 ml E*. coli* isolates whereas during the dry season, it was 8.66 $\times 10^{6}$ CFU/100 ml.

To explore whether the variations in *E. coli* counts exhibited in two different seasons were statistically significant or not, a paired *t*-test was conducted which required testing the normality of the data. The p-value for the normality test was 0.89 (is > 0.05) that satisfied the normality assumption. The p-value of the paired *t*-test was 0.94 which is greater than 0.05, so the variations among the *E. coli* counts seen in two seasons were not statistically significant at a 5 % level of significance.

For the isolation of ESBL *E. coli*, a maximum of 10 colonies were randomly picked from the mTEC agar plates for each of the 28 samples; a total of 266 isolates were taken randomly and inoculated on the CHROMagar™ ESBL agar by patch inoculation method. Phenotypically, 62 isolates were identified as ESBL producers in total among the 266 isolates and their prevalence was 23.31 % (62/266). The highest distribution (40 %) of ESBL *E. coli* was found in round 1 from WQ3 and in round 2 from WQ4, WQ5 and WQ7 during the wet season. The overall distributions of ESBL isolates for each sample are shown in Supplementary Table S1. The incidence of ESBL *E. coli* was higher in the wet season (62.90 %, 39/62) than observed in the dry season (37.10 %, 23/62). Out of 28 samples, ESBL *E. coli* contamination was found in 24 samples comprising 85.71 % (24/28) of the total samples.

### 2.2 A large number of ESBL producers contained *bla* genes

Among the four tested genes, *bla*_*CTX-M*_ turned out the most common in the 62 ESBL positive isolates and the percentage was 88.71 % (55/62), followed by *bla*_*TEM*_ in 27.42 % (17/62), *bla*_*SHV*_ in 1.61 % (1/62) and *bla*_*OXA*_ in 1.61 % (1/62). In addition, 58 were positive for at least one tested gene which is 93.55 % (58/62) of the total isolates. In addition, there were two combinations of co-existing genes in the isolates. One was CTX-M/TEM in 25.86 % (15/58) and the other was TEM/SHV in 1.72 % (1/58) of the isolates. The distribution of the *bla* genes was plotted in Fig. 3. Based on seasonal distribution, *bla*_*CTX-M*_ was present in 84.62 % (33/39) of the isolates of the wet season and 95.65 % (22/23) of the dry season. On the other hand, *bla*_*TEM*_ was in 17.95 % (7/39) and 43.48 % (10/23) of the isolates from wet and dry seasons respectively. Additionally, one isolate was positive for *bla*_*SHV*_ and *bla*_*OXA*_ genes each from the wet season. The *bla* genes present within each isolate is shown in Supplementary Table S3.

**Fig. 1:**
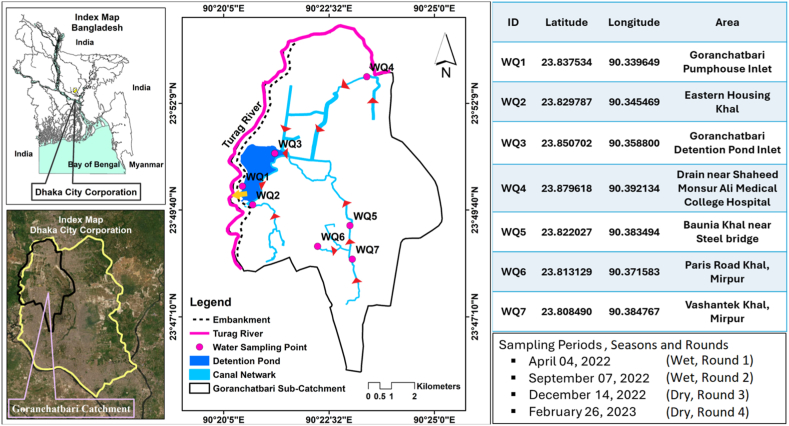
Geographical locations of the sampling points in Goranchatbari sub-catchment area. The table beside the map is showing latitudes and longitudes of the sampling locations; small box below includes the sampling periods and seasons as well. The red arrowheads on the map indicate the water flow pathways through the canals, while the yellow arrow shows the flow from the detention pond to the Turag river via the pump house.

**Fig. 2:**
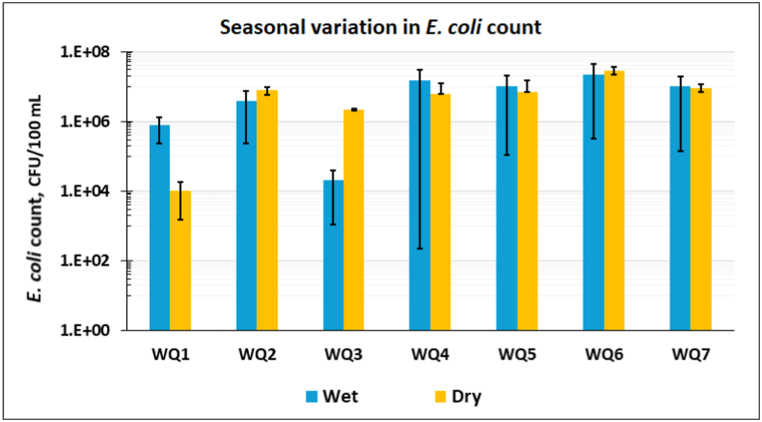
The average counts of *E. coli* for each sample with one standard deviation. The variations in counts observed here due to seasonality are not statistically significant (p > 0.05).

**Fig. 3:**
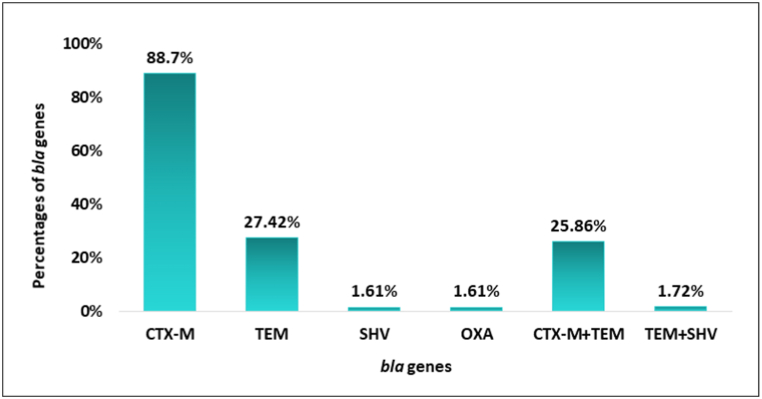
Distribution of different *bla* genes in ESBL *E. coli* isolates. The genes also co-exsisted in two combinations within 25.86 % and 1.72 % of the isolates as CTX-M + TEM and TEM + SHV. No isolates were positive for three or four genes combinedly.

### 2.3 Both diarrheagenic *E. coli* and ExPEC strains were present

The diarrheagenic multiplex PCR revealed that, among 62 ESBL isolates, 25 had a minimum of one out of the ten tested genes that comprised 40.32 % of the total isolates. One isolate was positive for *aaiC*, 22 were for *estA* and 5 for *eltB*. Among the diarrheagenic isolates, 4 % (1/25) were EAEC and 96 % (24/25) were ETEC. No other diarrheagenic pathotypes were found.

In the case of ExPEC, 37 non-diarrheagenic isolates were subjected to PCR analysis. Among the seven ExPEC associated genes, *sfaS* was the most prevalent and present in 62.16 % (23/37) of the isolates, followed by each of *iutA* and *kpsMII* in 16.22 % (6/37), *papA* and *afa* in 5.41 % (2/37), and *focG* in 2.70 % (1/37). Among the tested isolates, about 10.81 % (4/37) were positive for at least three of the seven ExPEC associated genes, thus considered as ExPEC strains as per recommendation [34]. Results are shown in Table 1. The presence of diarrheagenic and ExPEC associated genes within each isolates is listed in Supplementary Table S3.

**Table 1:** Prevalence of diarrheagenic and ExPEC isolates and the distribution of ExPEC associated genes.

**Pathogenicity**			
**Diarrheagenic**		**Non-diarrheagenic**	
25/62 (40.32 %)		37/62 (59.68 %)	
**Diarrheagenic pathotypes**		**ExPEC associated genes**	
ETEC	24/25 (96 %)	*focG*	01/37 (02.70 %)
		*kpsMII*	06/37 (16.22 %)
EAEC	1/25 (4 %)	*papA*	02/37 (05.41 %)
		*sfaS*	23/37 (62.16 %)
EPEC	0/25 (0 %)	*afa*	02/37 (05.41 %)
		*hlyD*	0/37 (0.0 %)
EHEC	0/25 (0 %)	*iutA*	06/37 (16.22 %)
EIEC	0/25 (0 %)	**ExPEC**	**4/37 (10.81 %)**

### 2.4 A substantial number of ESBL producers were MDR

**Fig. 4:**
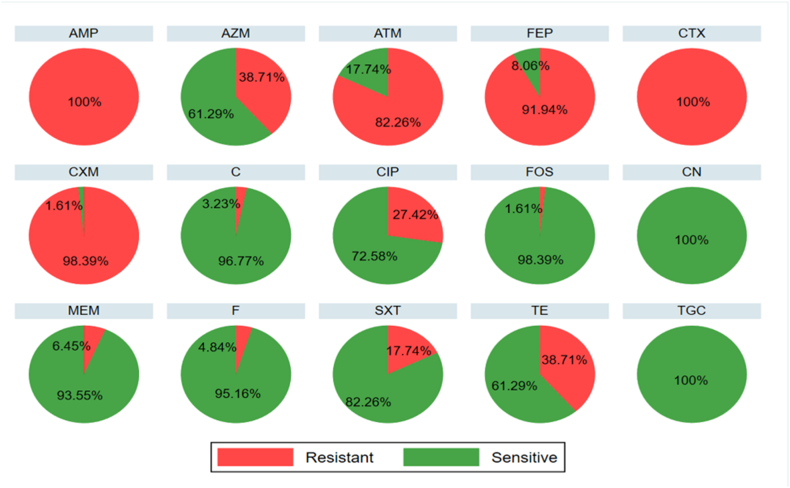
Antibiotic resistance profile of ESBL isolates highlighting the multidrug resistance potential of the isolates. The graphs are visually depicting the resistance and sensitivity percentages for individual antibiotic. All of the isolates were resistant to ampicillin and cefotaxime whereas were sensitive to gentamicin and tigecycline.

To investigate the resistance pattern of all the ESBL isolates against other antibiotics, antibiotic susceptibility testing (AST) was performed for 15 antibiotics from 15 different classes. All the 62 isolates were non-susceptible to ampicillin and cefotaxime. On the other hand, no resistance was found for tigecycline and gentamicin. All the isolates were MDR with the highest resistance recorded to 11 antibiotics for one isolate. About, 98.39 % (61/62) of the isolates were resistant to cefuroxime, followed by 91.94 % (57/62) to cefepime, 82.26 % (51/62) to aztreonam, 38.71 % (24/62) to tetracycline, 27.42 % (17/62) to ciprofloxacin, 17.74 % (11/62) to sulfamethoxazole-trimethoprim, 6.45 % (4/62) to meropenem, 4.84 % (3/62) to nitrofurantoin, 3.23 % (2/62) to chloramphenicol and 1.61 % (1/62) to fosfomycin. The overall antibiotic resistance pattern is presented in Fig. 4. All 62 isolates were MDR due to their resistance to at least 3 of the drug classes. The seasonal variations in resistance patterns of isolates to different antibiotics are shown in Table 2. The table revealed that there was no significant relationship between seasonal variation and resistance of isolates (p > 0.05), but at the level of 10 % significance, it was significant for SXT only. The resistance profiles of each isolate based on the zone diameter interpretation are given in Supplementary Table S3.

**Table 2:** Seasonal variations in antibiotic resistance patterns of ESBL *E. coli* isolates for different antibiotics with their statistical significance.

**Antibiotic resistance**			
**Antibiotics**	**Wet season** (N = 39)	**Dry season** (N = 23)	**P-value**
Ampicillin	100.00 %	100.00 %	1
Cefuroxime	97.44 %	100.00 %	1
Cefotaxime	100.00 %	100.00 %	1
Cefepime	87.18 %	100.00 %	0.14
Aztreonam	82.05 %	82.61 %	1
Tetracycline	30.77 %	52.17 %	0.11
Azithromycin	30.77 %	52.17 %	0.11
Ciprofloxacin	28.21 %	26.09 %	1
Sulfamethoxazole-trimethoprim	10.26 %	30.43 %	0.08
Meropenem	7.69 %	4.35 %	1
Chloramphenicol	2.56 %	4.35 %	1
Nitrofurantoin	7.69 %	0.00 %	1
Fosfomycin	2.56 %	0.00 %	1

### 2.5 ESBL positive isolates showed a higher tendency in biofilm formation at 25 °C

**Fig. 5:**
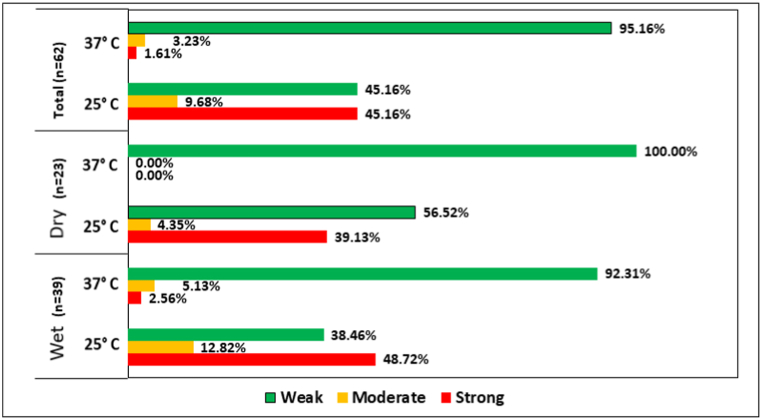
Distribution of ESBL biofilm former isolates with their biofilm forming capacity at two different temperatures. At 25 °C, isolates showed comparatively higher biofilm forming capacity in both wet and dry seasons.

The degree of biofilm formation demonstrated significant variations across different temperature conditions. Following a 48-h incubation period, in comparison to 37 °C, a substantial increase in overall biofilm formation became apparent at 25 °C. A large number of isolates from both dry and wet seasons showed strong biofilm formation at 25 °C. In contrast at 37 °C, most isolates were weak biofilm formers. At both temperatures, the number of strong biofilm formers was greater in the case of isolates from the dry season than in the wet. The percentages of the isolates for different biofilm forming capacities are shown in Fig. 5.

### 2.6 Genetic fingerprinting of the ESBL producers

**Fig. 6:**
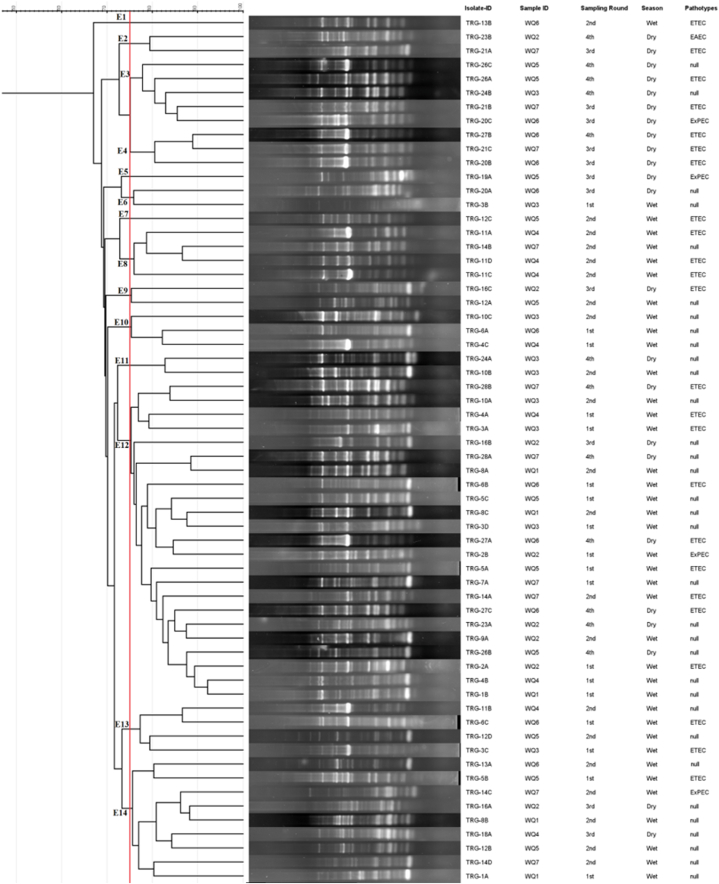
ERIC-PCR molecular fingerprint profiles of *E. coli* isolates with their representative ID, sampling point and round, and pathotypes. At 75 % similarity, the dendrogram grouped the isolates into 14 clusters.

Genetic fingerprinting of 62 ESBL isolates generated fingerprint bands where each pattern contained ten to twenty-one bands with a size variation from 75 to 2000 bp. The predominant bands were seen with sizes 700 bp and 1500 bp among the fingerprinting profiles of the isolates. Diverse banding patterns with 93 %–67 % similarity indices were revealed by the ERIC gel image analysis. At 75 % similarity index, the dendrogram generated 14 clusters (E1-E14) where the E12 was the largest containing 23 isolates from different sampling locations, rounds and seasons (Fig. 6). Among the isolates, two from the 1st round (TRG-1B and TRG-4B) shared 93 % genetic similarity with each other while the TRG-2A from another sampling point shared 89 % with them. On the other hand, TRG-28A and TRG-8A belong to two different sampling points from two different rounds and seasons shared ∼88 % similarity with each other. Among the ERIC profiles, three isolates (TRG-13B, TRG-19A and TRG-12C) were diverse, with genetic relatedness <75 % and single lineages. From the dendrogram, it was evident that the isolates irrespective of the sampling locations, rounds and seasons showed genetic similarities.

### 2.7 Relationship between the isolates’ genotypic and phenotypic characteristics

**Fig. 7:**
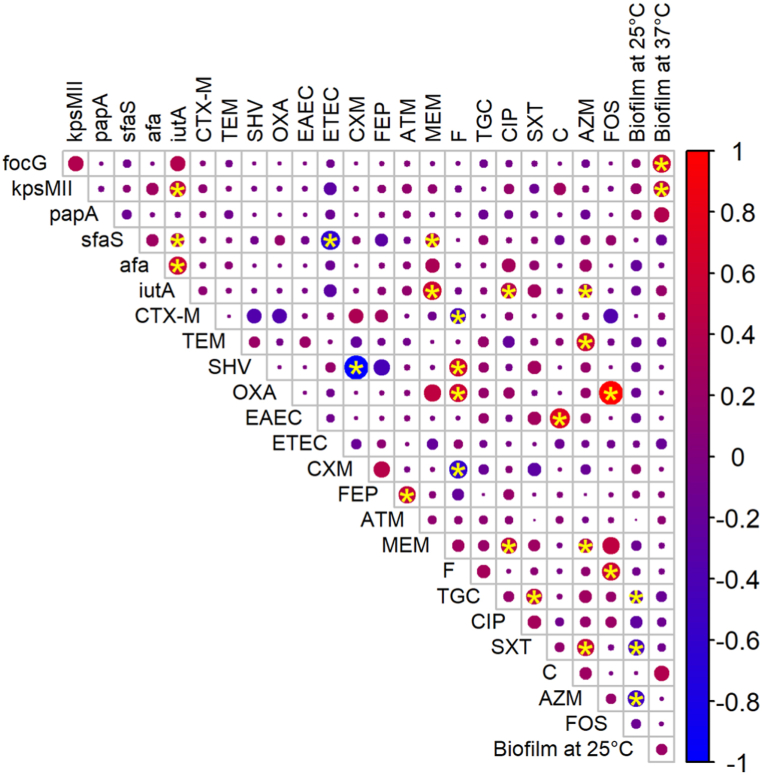
The association between the phenotypic and genotypic traits of the isolates. The correlation matrix of phenotypic traits (antibiotic resistance and biofilm formation ability) and genotypic traits (antibiotic resistance genes and virulence genes) displays the correlations. The size of the circles and intensity of the colors indicate the numerical values of the correlation coefficient where “Blue” refers to negative and “Red” to positive relationship. Significant associations (p < 0.05), as determined by Fisher's exact test, are marked with a "yellow star" symbol. Blue circles with “yellow star” sign indicated a significant negative correlation and red circles with “yellow star” sign showed a significant positive correlation. Spaces without “yellow star” sign are not significantly correlated.

The association between the phenotypic and genotypic traits of the isolates was explored using a correlation plot (Fig. 7). It revealed that there was a positive relationship between the distribution of *bla* genes and β-lactam resistance. Resistance to carbapenem and fosfomycin was positively correlated with the presence of *bla*_*OXA*_, and nitrofuran with the presence of *bla*_*SHV*_ and *bla*_*OXA*_. EAEC pathotype showed a significant correlation with chloramphenicol and a weak-positive correlation with SXT resistance. There was a significant correlation found between the capacity to develop biofilm at 37 °C and the presence of certain pathogenic genes like *focG* and *kpsmII*. Significant co-occurrence between *iutA*, *kpsmII*, *sfaS* and *afa* pathogenic genes was also observed. Along with this, co-resistance also occurred among different antibiotics such as cefuroxime and cefepime, meropenem and ciprofloxacin, meropenem and fosfomycin, and nitrofurantoin and fosfomycin.

A significant association was observed between the chloramphenicol resistance and bacterial biofilm formation capacity at 37 °C. Other than this, to some extent a relationship between biofilm formation and resistance to antibiotics was observed. For instance, among the isolates resistant to ampicillin, cefuroxime, cefotaxime, cefepime, aztreonam and chloramphenicol, about 50 % formed strong biofilm at 25 °C. Table 3 shows how the biofilm formation capacity was related to the antibiotic resistance.

**Table 3:** Association between ESBL *E. coli* isolates and their biofilm producing capacity.

**Antibiotics**	**Number of resistant isolates** **N**	**Biofilm at 25 °C**			**Biofilm at 37 °C**		
		**Weak biofilm producer n (%)**	**Moderate biofilm producer n (%)**	**Strong biofilm producer n (%)**	**Weak biofilm producer n (%)**	**Moderate biofilm producer n (%)**	**Strong biofilm producer n (%)**
Ampicillin	62	28 (45.16 %)	6 (9.68 %)	28 (45.16 %)	59 (95.16 %)	2 (3.23 %)	1 (1.61 %)
Cefuroxime	61	27 (44.26 %)	6 (9.84 %)	28 (45.90 %)	58 (95.08 %)	2 (3.28 %)	1 (1.64 %)
Cefotaxime	62	28 (45.16 %)	6 (9.68 %)	28 (45.16 %)	59 (95.16 %)	2 (3.23 %)	1 (1.61 %)
Cefepime	57	25 (43.86 %)	5 (8.77 %)	27 (47.37 %)	54 (94.74 %)	2 (3.51 %)	1 (1.75 %)
Aztreonam	51	23 (45.10 %)	5 (9.80 %)	23 (45.10 %)	48 (94.12 %)	2 (3.92 %)	1 (1.96 %)
Tetracycline	24	15 (62.50 %)	8 (33.33 %)	1 (4.17 %)	24 (100.00 %)	0 (0.00 %)	0 (0.00 %)
Azithromycin	24	18 (75.00 %)	0 (0.00 %)	6 (25.00 %)	23 (95.83 %)	1 (4.17 %)	0 (0.00 %)
Ciprofloxacin	17	11 (64.71 %)	1 (5.88 %)	5 (29.41 %)	17 (100.00 %)	0 (0.00 %)	0 (0.00 %)
Sulfamethoxazole-trimethoprim	11	10 (90.91 %)	0 (0.00 %)	1 (9.09 %)	11 (100.00 %)	0 (0.00 %)	0 (0.00 %)
Meropenem	4	3 (75.00 %)	0 (0.00 %)	1 (25.00 %)	4 (100.00 %)	0 (0.00 %)	0 (0.00 %)
Chloramphenicol	2	1 (50.00 %)	0 (0.00 %)	1 (50.00 %)	1 (50.00 %)	1 (50.00 %)	0 (0.00 %)
Nitrofurantoin	3	2 (66.67 %)	0 (0.00 %)	1 (33.33 %)	3 (100.00 %)	0 (0.00 %)	0 (0.00 %)
Fosfomycin	1	1 (100.00 %)	0 (0.00 %)	0 (0.00 %)	1 (100.00 %)	0 (0.00 %)	0 (0.00 %)

## 3 Discussion

Being the capital of Bangladesh and a densely populated megacity, Dhaka faces a huge amount of untreated domestic and industrial wastewater discharge into the surface water bodies due to insufficient treatment facilities. Previous studies on surface water quality did not include water bodies like drains and detention ponds connected to the river. This study aimed at analyzing water from seven different locations of the Goranchatbari sub-catchment area for ESBL *E. coli*, their resistance patterns and pathogenicity, distribution of resistance genes and clonal relationship seasonally. In Bangladesh, a prominent reason for surface water contamination is the disposing of household wastes, cattle washing, connecting to sewers, opening of latrines and industrial waste into the water bodies. Owing to these, the majority of the water reservoirs of the country are becoming hazardous for human, animal and aquatic life [35]. Understanding these pollution sources is crucial for devising effective water management strategies.

In this study, a substantial number of *E. coli* were found in all the water samples irrespective of the seasonal variation. All the sites, water bodies were receiving wastewater from different sources, so it is obvious why all of them were contaminated with *E. coli*. Among the sampling points, WQ5, WQ6 and WQ7 showed higher counts throughout the seasons with an ignorable variation because of being khals that receive domestic wastewater from the surrounding areas as well as direct disposal of latrine waste throughout the year. The average count of *E. coli* was slightly higher in the wet season than in the dry, aligning with some previous studies [[36], [37], [38]]. This might be due to the contaminated runoffs from the surface and during the dry season, the water level goes down in the water bodies keeping the degree of contamination nearly similar. Additionally, low temperature and moisture content in the dry season may also have contributed to the decreased number of *E. coli*.

ESBLs are of great microbiological and clinical importance in *E. coli*. In surface water, the occurrence of ESBL *E. coli* has been reported previously globally which signified that the presence of ESBL genes in the bacteria in turn pointed to the presence of ESBL in the population [39,40]. *E. coli* has become a serious health concern to both humans and animals. Due to the widespread use of β-lactam antibiotics, *E. coli* has developed resistance, leading to widespread hard-to-treat diseases in the communities [41]. Infections caused by ESBL-producing *E. coli* present a considerable threat to healthcare settings due to the limited availability of effective empirical treatments [42]. In the current study, the samples were also contaminated with ESBL *E. coli*; among 28 samples, about 85.71 % were found positive for the presence of ESBL *E. coli*. Previously a study conducted in rural areas of Bangladesh revealed that 76 % of the pond, 85 % of the river and 90 % of the wastewater (adjacent to household or poultry farm wastewater drain) samples were contaminated with ESBL *E. coli* [43]. The prevalence of ESBL *E. coli* isolates was 23.31 % in the present study. In another study conducted in Bangladesh, about 37.5 % of the *E. coli* in surface water samples collected from eight points of two rivers and two lakes around Dhaka city were reported to be positive for ESBL phenotype [24]. These findings highlight the pervasive nature of ESBL *E. coli* in various water sources, emphasizing the need for continuous monitoring.

One prominent mechanism of evading the action of extended spectrum β-lactams is through various ESBL genes. The most prominent types of ESBL enzymes include TEM, SHV and CTX-M [44]. The OXA-type enzymes are another growing family of ESBLs, unique among them for their potential to inactivate a broader range of β-lactam antibiotics due to their class D origin and wider substrate profiles [13,45]. It is crucial to know the underlying genes responsible for encoding ESBLs within the isolates. In our study, we detected *bla*_*CTX-M*_ in 88.71 % of the isolates, followed by *bla*_*TEM*_ in 27.42 %, *bla*_*SHV*_ in 1.61 % and *bla*_*OXA*_ in 1.61 %. In a study conducted in the Rohingya camps on fecal sludge wastewater, 93 % of isolates contained *bla*_*CTX−M−1*_ gene, followed by 58 % *bla*_*CTX−M−15*_, 42 % *bla*_*TEM*_ and 2 % *bla*_*SHV*_ genes [34]. A similar pattern was also found in our study. Another study conducted on waste and surface waters reported an increased incidence of *bla*_*CTX-M*_, followed by *bla*_*TEM*_*, bla*_*OXA*_ and *bla*_*SHV*_ genes [46]. The possible reason for the decreased prevalence of *bla*_*OXA*_ may be due to being environmental isolates [47]. In different parts of the world, it was shown that *bla*_*CTX-M*_ was widespread among *E. coli* [48,49]. The wastewater is considered the reservoir of ESBL genes and the positive pressures on the bacteria present over there may include contact with residual antimicrobials that in turn contribute to the development and transfer of resistance [43,50]. These findings underscore the importance of identifying and mitigating the sources of antimicrobial residues in the environment to combat the spread of resistance genes.

*E. coli* could potentially be an extensively versatile and often fatal pathogen in addition to being a harmless intestinal inhabitant. Certain strains of *E. coli* use virulence factors that alter a broad range of biological processes triggering a variety of intestinal and extraintestinal diseases [15]. In our study, we found 40.32 % diarrheagenic *E. coli* among which 96 % were ETEC and 4 % EAEC. This might be due to the discharge of human excreta in the water bodies of that area. This finding is in concordance with another study where ETEC was detected from surface water in rural and urban areas of Bangladesh [51]. In another study, ETEC was isolated from different rivers around Dhaka city [52]. Azmuda N et al. showed that EPEC and ETEC were more prevalent than other pathotypes of *E. coli* in Brahmanbaria pond and Dhanmondi lake water of Bangladesh, but EAEC was occasionally recovered from the pond only [53]. ETEC strains are linked to two primary clinical conditions: weanling diarrhea in children in developing countries and traveler's diarrhea. Typically, they attach themselves to intestinal epithelial cells via colonization factors (CFs), a diverse collection of protein-based surface structures, which can be fimbrial, non-fimbrial or fibrillar [54]. In Bangladesh, ETEC is a frequent cause of acute watery diarrhea in infants and young children, accounting for approximately 20 % cases of diarrhea in children under two years old [55,56]. Studies indicate that diarrhea affects 20 %–60 % of tourists, with ETEC responsible for 20 %–40 % of these cases [15]. EAEC ranks second as a reason for travelers' diarrhea globally, following ETEC, affecting both developed and developing countries. In developed countries, it has been shown to induce acute diarrheal sickness in infants and young children, and is linked to chronic diarrhea. Diarrhea caused by EAEC is typically watery but may also present with mucus or blood. During the pathogenesis of EAEC, the intestinal mucosa becomes colonized, mucoid biofilms form, several enterotoxins and cytotoxins are produced, and mucosal inflammation occurs [57]. In a study conducted in Rohingya camps' fecal sludge wastewater, both ETEC and EAEC were detected at a percentage of 75 % and 13 % respectively [34]. Diarrheagenic *E. coli* from the sub-catchment area may migrate to the adjacent Turag river, potentially contaminating surrounding water bodies along its course and increasing the risk of infection for the local population. These findings illustrate the significant health risks posed by pathogenic *E. coli* in contaminated water sources, highlighting the need for improved sanitation and water treatment practices.

Both humans and animals can contract a range of diseases from ExPEC, such as septicemia, meningitis, and urinary tract infections (UTIs). They are different in terms of their epidemiology and genetic lineage compared to both commensal and intestinal pathogenic strains. These strains can effectively colonize the host's intestinal tract and are found as the predominant strain in approximately 20 % of healthy human hosts [17]. Among the tested isolates, we detected 10.81 % as ExPEC strains. Previously, a comparable incidence of about 11 % of ExPEC isolates was reported in sewage and environmental samples which supports our findings [58]. On the other hand, in fecal wastewater of Rohingya camps, 3 % ExPEC isolates were detected [34]. In addition to this, a few other studies also reported the recovery of ExPEC from environmental water samples in different parts of the world [[59], [60], [61]]. These data suggest that ExPEC strains are prevalent in various water environments, posing a chance for the spread of serious infections to humans and animals.

The overuse or abuse of antibiotics, and the presence of residual antimicrobials and heavy metals in wastewater, further exacerbate the issue, triggering a positive pressure on the microbial community living there, resulting in the development of antibiotic resistance [43,62,63]. Infection with resistant bacteria severely limits the existing therapeutic options, a consequence that would be fatal because for *E. coli*, currently there is no vaccination available [64]. In the present study, we determined the antibiotic susceptibility profiles of all 62 ESBL isolates, providing insight into their multidrug resistance potential. In our study, high resistance was observed in the case of β-lactam antibiotics which included ampicillin, cefotaxime, cefuroxime, cefepime and aztreonam. This might be due to the selection of ESBL positive isolates which can hydrolyze a wide array of β-lactams [65]. Another striking finding is that all the isolates were MDR, resistant to at least three drug classes. The reason behind this might be resistance to other antibiotics was co-selected within the ESBLs. Moreover, all the sampling sites are connected to drainage systems that contain wastes from hospitals and households. Antibiotics, ARB, and MDR bacteria are among such waste materials that are found in surface waters contaminated in this way [66]. A high abundance of MDR *E. coli* has been recorded in the surface and wastewater of Bangladesh and other parts of the world [24,34,46,67]. One well-known MDR pathogen that has been involved in severe hospital and community-acquired infections globally is *E. coli* strains that produce ESBLs [68]. These findings underscore the need for stringent regulations on antibiotic use and improved wastewater treatment processes to mitigate the spread of resistant bacteria.

Another key aspect to consider is that *E. coli* is a well-known biofilm former on both biotic and abiotic surfaces. Biofilm formation significantly contributes to bacterial infection persistence, enhancing resistance to treatment and disinfection [69]. Bacteria that form biofilms commonly cause recurrent and complicated UTIs, often involving MDR strains [70]. In our study, the tested isolates showed different degrees of biofilm formation at 37 °C and 25 °C, ranging from weak to strong. The water from the Goranchatbari sub-catchment area is pumped out to the adjacent Turag river, creating a potential health threat to the people living in that area resulting from the contamination of the surface water with the biofilm former strains. Consequently, this contamination may be transmitted to the drinking water of the adjacent area. However, bacterial biofilm formation may also aid the process of wastewater treatment. Biofilms based wastewater treatment technologies are employed to eliminate pollutants from wastewater, including nitrogenous and organic materials. Different types of chemical mediators involved in biofilm induced quorum sensing from a variety of bacterial species play roles in environmental bioremediation such as remediation of toxic contaminants in wastewater and soil [71]. Hence, biofilm formation presents both challenges and opportunities in managing water quality.

Investigating the molecular diversity of bacteria, several methods exist e.g. amplified fragment length polymorphism, amplified ribosome DNA restriction analysis, random amplified polymorphism DNA, ribotyping, restriction fragment length polymorphism etc. Of the mentioned methods, ERIC-PCR is rapid, user-friendly, and cost-effective, yielding satisfactory results. It is known for its reliable discriminatory power as well as its swift and relatively straightforward technique, making it valuable for routine epidemiological studies. However, its reproducibility is low [72]. In this study, at 75 % similarity, the dendrogram generated 14 clusters with the largest cluster E12 containing 23 isolates from different sampling locations, time frames and different pathotypes. Among our isolates, only two (TRG-1B and TRG-4B) were considered to be genetically related as their similarity rate was above 90 % [73]. Other isolates revealed a genetic similarity below 90 % among themselves and hence were considered genetically unrelated. This data indicated a complex and diverse fingerprint variation among the isolates. Similar diverse *E. coli* have been found in surface and wastewater previously [34,74,75]. This variation might be due to the mutations in the genomes taking place while persisting in the environment [76]. Thus, genetic diversity among isolates is significant for understanding the spread of resistance.

Interestingly, a significant association was observed between ExPEC associated virulence genes, resistance to different antibiotics and biofilm formation capacity. Previously, it has been shown that some ExPEC genes are also associated with biofilm formation [77]. We also found a correlation between the existence of resistance genes and antibiotic resistance; this finding is also similar to previous studies [34,47,78]. For the β-lactam antibiotics, we found ∼50 % of the resistant isolates were strong biofilm formers at 25 °C (Table 3). Previous research has also suggested that bacteria capable of forming biofilms often display greater resistance compared to planktonic cells, primarily due to the hard polymeric matrix that hinders antibiotic penetration [79]. Extracellular polymeric components of biofilm can suppress the action of pesticides, hydrocarbons, and heavy metals which are present in the immediate surrounding environment of biofilm [80]. These findings underscore the complexity of addressing biofilm-associated antibiotic resistance.

The findings of this study highlight significant public health concerns associated with the presence of MDR ESBL-producing *E. coli* in urban surface waters of Dhaka. The detection of high levels of ESBL *E. coli*, including pathogenic strains such as ETEC and ExPEC, underscores the potential risk of waterborne diseases and the spread of AMR in the community. Contaminated surface waters can act as reservoirs and transmission pathways for these resistant pathogens, posing a direct threat to human health.

This study has a few limitations. Firstly, the study lacks plasmid information of the isolates and the conjugation ability testing which limits the insight of the transferability of the mobile genetic element. Secondly, though PCR revealed important information about resistance, we did not conduct any PCR for other CTX-M lineages and did not sequence the amplicons of the ESBL genes. Despite these limitations, this study provides detailed insights into the resistance patterns of ESBL *E. coli* isolated from surface water, their pathogenic makeups, and the association between different parameters, which necessitates the importance of further studies on the surface water of that area. Future research should focus on overcoming these limitations to enhance our understanding of antibiotic resistance mechanisms.

## 4 Conclusion

Water resources are often subjected to pollution from municipal wastes, leading to pathogenic bacterial contamination and directly affecting the prevalence of waterborne diseases in humans. This study investigated the microbial contamination of the surface water in the Goranchatbari sub-catchment area of Dhaka city. A high prevalence of *E. coli* in the surface water with the presence of MDR pathogenic ESBL isolates were found. These isolates pose a public health threat by potentially spreading AMR to adjacent areas through the water flow of the Turag river as the river travels a significant distance, connecting with other water bodies and communities along its course. Additionally, pisciculture was noticed within the detention area which can be a human health safety issue for the local people. AMR may also affect the aquatic species such as fishes in the Turag river and find their way to the food chain. Thus, it is possible that the ESBL-producing Enterobacteriaceae in the surface waters of Bangladesh could play a role in the dissemination of antibiotic resistant infections both in humans and animals. Therefore, it is essential to closely study, through detailed epidemiological research, how the environmental and clinical bacterial isolates interact. This approach is necessary to comprehend both existing and emerging MDR infections. Antibiotic therapies that are not required must be avoided, and uncontrolled therapies ought to be restricted. In conclusion, it might be stated that the water quality was insufficient to safeguard public health and emphasizes the need for effective management to achieve optimal ecosystem balance and ensure high water quality. Effective control strategies are highly recommended in the vicinity of the studied region for the protection and improvement of public health outcomes.

## 5 Materials and methods

### 5.1 Sampling site selection

Among the 10 sub-catchment areas of western Dhaka city- Goranchotbari, Rampura, Kallyanpur, Dholaikhal, Kamalapur, Nawabgonj, Shahidnagar, Sadarghat, Kamalbagh, and Basabo, the largest Goranchatbari (67.5 square kilometers) was selected to investigate the microbiological quality of certain types of surface water bodies e.g. khals, drains and detention ponds. In Goranchatbari, there is a detention pond (2.6 square kilometers) that receives stormwater and wastewater from the entire sub-catchment area through khals; the accumulated water is then pumped out to the adjacent Turag river thus affecting the water quality of the river. Within the whole sub-catchment area, seven distinct severed waterbody sites were selected for sample collection. The latitudes and longitude of sampling sites are given in Fig. 1.

### 5.2 Sampling procedure and periods

Samples were collected from the seven selected sites of the Goranchatbari sub-catchment in Dhaka city (Fig. 1). The sites included canals, local drains and detention ponds or wetlands. The figure also shows an environmental transmission pathway from drains – canals – detention pond – discharge into river.

Samples were collected in four rounds during the months of April, September, December in 2022 and February in 2023. From the climatic perspective of Bangladesh, these can be defined as pre-monsoon (April), monsoon (September), post-monsoon (December) and dry (February) seasons. For this study, these can be grouped as Wet season (April and September) and Dry season (December and February). Approximately, 500 mL of composite sample was collected in sterile high-density polyethylene (HDPE) plastic bottles (NALGENE, NY, USA) with proper labels. During collection, composite samples were taken for a better representative sample of the stream-at least 100 m away from any junction of multiple streams and approx. 2.5 m from the canal bank. All the samples were collected from a depth of about 15 cm below the water surface. Following collection, the samples were sent in an insulated box with a temperature range of 4 °C–10 °C to the Laboratory of Environmental Health, icddr,b, Dhaka and processed on the same day [81,82].

### 5.3 Processing the samples and isolating ESBL *E. coli*

Before processing, each sample was assigned a unique lab ID and brought to room temperature, shaken thoroughly for a uniform mixture. Briefly, following mixing, 25 μl from undiluted as well as each of 3 decimal serial dilutions (10^−1^ to 10^−3^) of every sample was inoculated based on previous experience for countable colonies using drop-plate inoculation technique on mTEC (Modified Thermotolerant *E. coli*) agar plates (BD Difco, NJ, USA) [83]. Subsequently, the plates underwent incubation for 2 h at 37 ± 0.5 °C, and then again for another 22–24 h at 44 ± 0.5 °C. Colonies that turned magenta following incubation were regarded as possible *E. coli*. Colonies from the countable dilution were multiplied by the dilution factor to express the counts as CFU/100 ml. For the subsequent screening and isolation of ESBL producing *E. coli*, a maximum of ten colonies were selected randomly from each sample and screened following a previously published protocol using the CHROMagar™ ESBL (CHROMagar, Paris, France) [34]. Additionally, ESBL *E. coli* CIP 103982 was used as the positive control and *E. coli* ATCC 25922 as the negative control. Each ESBL isolate from a sample was assigned a unique ID, which began with the sample's lab ID followed by a letter in ascending order from 'A' onward if there were multiple isolates from the same sample. Additionally, the isolates were verified using the API 20E kit (Biomerieux SA, Marcy-I'Etoile, France) following the manufacturer's instructions.

### 5.4 Identification of antibiotic resistance associated genes

Following the previously published protocol of the boiling lysis method, DNA extraction was carried out from all the phenotypically confirmed ESBL *E. coli* isolates [84]. Next, to find out if there were any antibiotic resistance genes in the isolates, PCR analysis was performed with the DNA contents for *bla*_*SHV*_, *bla*_*TEM*_, *bla*_*CTX-M*_ and *bla*_*OXA*_, following the protocol outlined previously [85]. Positive controls for these reactions were sourced from earlier studies [47]. The primer sequences and the respective product sizes are detailed in Supplementary Table S2. Subsequent to the PCR, the products were subjected to horizontal electrophoresis using 1 % agarose gel and 0.5X tris borate EDTA buffer. The GelDoc Go Imaging System (BIORAD, California, USA) was then used to visualize the gels and take the images.

### 5.5 Detection of *E. coli* pathotypes

For the identification of the presence of diarrheagenic genetic determinants in all the ESBL producers, molecular analysis was performed following a previously published protocol [84]. For this purpose, PCR was done for the following genes e.g. anti-aggregation protein transporter (*aat*) and aggR-activated island (*aaiC*) for EAEC, attaching and effacing (*eae*) and bundle forming pilus (*bfp*) for EPEC, heat-labile (*lt*) and heat-stable (*st*) for ETEC, invasion plasmid antigen H (*ipaH*) and the invasion-associated locus (*ial*) for EIEC [34]. For EHEC, the Shiga toxin encoding virulence factors (*stx1* and *stx2*) were detected using PCR [34].

Additionally, seven genes related to the ExPEC were searched for in the remaining non-diarrheagenic isolates. The virulence factors are *afa* (afimbrial adhesins), *focG* (F1C fimbriae), *hlyD* (cytolytic protein toxin), *iutA* (iron acquisition system), *kpsMII* (group 2 polysaccharide capsule), *papA* (P fimbriae), and *sfaS* (S fimbriae). To determine the ExPEC related genes, two distinct multiplex PCRs were conducted, following the protocol published before [34]. Positive controls were adapted from a previously published study [47]. The primer sequences, their corresponding product sizes, and associated pathotypes are provided in Supplementary Table S2.

### 5.6 Determining the patterns of antibiotic resistance in ESBL producers

The antibiotic susceptibility profiles of all ESBL isolates were determined according to the guidelines of the Clinical Laboratory Standards Institute (CLSI) and the European Committee on Antimicrobial Susceptibility Testing (EUCAST), using the Kirby-Bauer disk diffusion method as recommended [[86], [87], [88]]. The isolates were tested for susceptibility against 15 different antibiotics, each representing a different class. Disks available commercially (BioMaxima S.A., Lublin, Poland) were used for the following antibiotics e.g. ampicillin (10 μg, AMP), cefuroxime (30 μg, CXM), cefotaxime (30 μg, CTX), cefepime (30 μg, FEP), aztreonam (30 μg, ATM), meropenem (10 μg, MEM), gentamicin (10 μg, CN), nitrofurantoin (300 μg, F), tetracycline (30 μg, TE), tigecycline (15 μg, TGC), ciprofloxacin (5 μg, CIP), sulfamethoxazole-trimethoprim (25 μg, SXT), chloramphenicol (30 μg, C), azithromycin (15 μg, AZM) and fosfomycin (200 μg, FOS). *E. coli* ATCC 25922 was used for QC purposes in this assay and the interpretation for all antibiotics except tigecycline was made following the CLSI guideline, and the EUCAST guideline was used to interpret tigecycline [87,88]. It is important to note that all the intermediate-resistant were considered resistant in this study.

### 5.7 Determination of biofilm formation capacity

Using the quantitative adherence assay the capacity of the ESBL isolates to produce biofilms was evaluated [47]. Briefly, a fresh enrichment culture of the isolate was made and inoculated in a 96-well plate at a 100-fold dilution. Biofilm formation capacity was tested at two different temperatures, 25 °C and 37 °C, with an incubation period of 48 h. For QC purposes, necessary blanks within each plate and assay duplications at each temperature were performed. The microtiter plate's optical density (OD) was assessed using an ELISA plate reader (BioTek, Vermont, USA) at a wavelength of 590 nm after the biofilm had formed. According to a previously established protocol, the isolates were classified as strong, moderate, weak, or non-biofilm formers based on their degree of biofilm formation [89].

### 5.8 Genetic fingerprinting of ESBL *E. coli* through ERIC

An Enterobacterial Repetitive Intergenic Consensus Polymerase Chain Reaction (ERIC-PCR) was employed to assess the genetic similarity among the ESBL isolates in accordance with a previously described procedure [78]. The amplicons were resolved in 2 % agarose gel with 1X TBE where Thermo Scientific™ GeneRuler 1 kb Plus DNA Ladder (Thermo Fisher Scientific Baltics UAB, Vilnius, Lithuania) was used. Applying the dice coefficient and the unweighted-pair group method using average linkages (UPGMA), the GelJ v.2.0 software [90] analyzed the gel images after normalizing them via the gaussian regression method to produce dendrograms with 1.0 % tolerance levels. Based on the genetic distances measured between isolates, the UPGMA algorithm constructs a hierarchical tree.

### 5.9 Statistical analysis

The statistical analyses were conducted using the Microsoft Worksheet (Excel 2019), R programming (version 4.3.2) and Stata 15. To determine the significance of *E. coli* count variation, a paired *t*-test was performed. For profiling the presence of resistance and virulence genes, all the data were transformed to binary format, where a value of 1 indicated the examined genes were present and a value of 0 indicated their absence. Additionally, the binary format was utilized for the interpretation of the antibiogram data in Excel, R programming and Stata 15. The significance of seasonal variation among the antibiotic resistance patterns was evaluated using the Fisher exact test. The correlations between different variables were achieved via ‘cor’ function and the significance (p < 0.05) determination was conducted by employing the ‘fisher.test’ function using the Fisher exact correlation test. The significant correlations between the variables, i.e. phenotypic and genotypic traits, were shown utilizing the ‘corrplot’ function [47,85]. All the isolates demonstrated a 100 % resistance to ampicillin and cefotaxime, and all were sensitive to tigecycline and gentamicin. Hence, the correlation plot excluded these four variables since their correlation showed no obvious significance.

## CRediT authorship contribution statement

**Md. Sakib Hossain:** Writing – review & editing, Writing – original draft, Visualization, Validation, Methodology, Formal analysis, Data curation, Conceptualization. **Ahmed Ishtiaque Amin Chowdhury:** Writing – review & editing, Visualization, Validation, Supervision, Project administration, Investigation, Funding acquisition, Conceptualization. **Mohammad Rafiqul Islam:** Writing – review & editing, Validation, Methodology, Investigation. **Ripan Kirtunia:** Writing – review & editing, Validation, Methodology, Conceptualization. **Md. Foysal Abedin:** Writing – review & editing, Visualization, Software, Formal analysis, Data curation. **Mohammad Atique Ul Alam:** Writing – review & editing, Validation. **Shampa:** Writing – review & editing, Validation. **Sonia Binte Murshed:** Writing – review & editing, Validation. **Md Shadman Sakib:** Writing – review & editing, Validation. **Siam Alam:** Writing – review & editing, Validation. **M. Shahjahan Mondal:** Writing – review & editing, Validation, Resources, Conceptualization, Project administration, Funding acquisition. **Zahid Hayat Mahmud:** Writing – review & editing, Validation, Supervision, Resources, Project administration, Investigation, Funding acquisition, Conceptualization.

## Ethics statement

The work described has not been published previously, and it is not under consideration for publication elsewhere, in full or in part. The publication of this article is approved by all authors. This article does not contain any studies with human participants or animals performed by any of the authors.

## Data availability statement

Data associated with this study has not been deposited into a publicly available repository. Data will be made available on request.

## Funding

This research was funded by the U.S. Department of State through 10.13039/501100009500BUET under the project “Assessing Health Impacts of Urban Flooding under Changing Climate: A Case Study in Dhaka City”.

## Declaration of competing interest

The authors declare that they have no known competing financial interests or personal relationships that could have appeared to influence the work reported in this paper.
