# Supplementary material for: Multidrug-resistant ESBL E. coli in urban surface waters and public health implications: A Case Study from Goranchatbari, Dhaka
Source: Heliyon. 2025 Jan 23;11(3):e42219. doi: 10.1016/j.heliyon.2025.e42219 (PMC11815672; doi:10.1016/j.heliyon.2025.e42219)
Supplement: Multimedia component 1 [file mmc1.docx]

**Supplementary table S1:** Lab ID, counts of *E. coli* and the percentage of ESBL *E. coli* in each sample.

| **Sl. No.** | **Lab ID** | **Name of Sample** | **Sampling Round** | **Sampling season** | ***E. coli* (CFU/100ml)** | **Percentage of ESBL *E. coli*** |
| --- | --- | --- | --- | --- | --- | --- |
|  | **TRG-1** | WQ1 | 1^st^ | Wet | 232000 | 20.00% |
|  | **TRG-2** | WQ2 | 1^st^ | Wet | 232000 | 20.00% |
|  | **TRG-3** | WQ3 | 1^st^ | Wet | 40000 | 40.00% |
|  | **TRG-4** | WQ4 | 1^st^ | Wet | 221 | 30.00% |
|  | **TRG-5** | WQ5 | 1^st^ | Wet | 108000 | 30.00% |
|  | **TRG-6** | WQ6 | 1^st^ | Wet | 332000 | 30.00% |
|  | **TRG-7** | WQ7 | 1^st^ | Wet | 144000 | 10.00% |
|  | **TRG-8** | WQ1 | 2^nd^ | Wet | 1320000 | 30.00% |
|  | **TRG-9** | WQ2 | 2^nd^ | Wet | 7600000 | 10.00% |
|  | **TRG-10** | WQ3 | 2^nd^ | Wet | 1100 | 30.00% |
|  | **TRG-11** | WQ4 | 2^nd^ | Wet | 29600000 | 40.00% |
|  | **TRG-12** | WQ5 | 2^nd^ | Wet | 20000000 | 40.00% |
|  | **TRG-13** | WQ6 | 2^nd^ | Wet | 44000000 | 20.00% |
|  | **TRG-14** | WQ7 | 2^nd^ | Wet | 19600000 | 40.00% |
|  | **TRG-15** | WQ1 | 3^rd^ | Dry | 4000 | 0.00% |
|  | **TRG-16** | WQ2 | 3^rd^ | Dry | 6360000 | 30.00% |
|  | **TRG-17** | WQ3 | 3^rd^ | Dry | 2280000 | 0.00% |
|  | **TRG-18** | WQ4 | 3^rd^ | Dry | 1600000 | 10.00% |
|  | **TRG-19** | WQ5 | 3^rd^ | Dry | 920000 | 10.00% |
|  | **TRG-20** | WQ6 | 3^rd^ | Dry | 33600000 | 30.00% |
|  | **TRG-21** | WQ7 | 3^rd^ | Dry | 7600000 | 30.00% |
|  | **TRG-22** | WQ1 | 4^th^ | Dry | 16000 | 0.00% |
|  | **TRG-23** | WQ2 | 4^th^ | Dry | 9200000 | 20.00% |
|  | **TRG-24** | WQ3 | 4^th^ | Dry | 2120000 | 20.00% |
|  | **TRG-25** | WQ4 | 4^th^ | Dry | 10400000 | 0.00% |
|  | **TRG-26** | WQ5 | 4^th^ | Dry | 12800000 | 30.00% |
|  | **TRG-27** | WQ6 | 4^th^ | Dry | 23600000 | 30.00% |
|  | **TRG-28** | WQ7 | 4^th^ | Dry | 10800000 | 20.00% |
