# Supplementary material for: Multidrug-resistant ESBL E. coli in urban surface waters and public health implications: A Case Study from Goranchatbari, Dhaka
Source: Heliyon. 2025 Jan 23;11(3):e42219. doi: 10.1016/j.heliyon.2025.e42219 (PMC11815672; doi:10.1016/j.heliyon.2025.e42219)
Supplement: Multimedia component 2 [file mmc2.docx]

**Supplementary table S2:** Primer sequences and amplicon sizes for antibiotic-resistance genes, virulent genes and their associated pathotypes

|  | **Target**  **gene** | **Primer**  **name** | | **Sequence**  **(5’ 🡪 3’)** | **Product**  **size (bp)** | **Reference** |
| --- | --- | --- | --- | --- | --- | --- |
| **ESBL primers** | *bla_SHV_* | SHV-F | | CTTTATCGGCCCTCACTCAA | 237 | [1] |
|  |  | SHV-R | | AGGTGCTCATCATGGGAAAG |  |  |
|  | *bla_TEM_* | TEM-F | | CGCCGCATACACTATTCTCAGAATGA | 445 |  |
|  |  | TEM-R | | ACGCTCACCGGCTCCAGATTTAT |  |  |
|  | *bla_CTX-M_* | CTX-M-F | | ATGTGCAGYACCAGTAARGTKATGGC | 593 |  |
|  |  | CTX-M-R | | TGGGTRAARTARGTSACCAGAAYCAGCGG |  |  |
|  | *bla_OXA_* | OXA-F | | ACACAATACATATCAACTTCGC | 813 |  |
|  |  | OXA-R | | AGTGTGTTTAGAATGGTGATC |  |  |
| **Intestinal pathogenic primers** | *estA* | ST-F | | GCTAAACCAGTA^G^_A_GGTCTTCAAAA | 147 | [2] |
|  |  | ST-R | | CCCGGTACA^G^_A_GCAGGATTACAACA |  |  |
|  | *eltB* | LT-F | | CACACGGAGCTCCTCAGT C | 508 |  |
|  |  | LT-R | | CCCCCAGCCTAGCTTAGTTT |  |  |
|  | *bfpA* | bfpA-F | | GGAAGTCAAATTCATGGGGG | 300 |  |
|  |  | bfpA-R | | GGAATCAGACGCAGACTGGT |  |  |
|  | *eae* | eae-F | | CCCGAATTCGGCACAAGCATAAGC | 881 |  |
|  |  | eae-R | | CCCGGATCCGTCTCGCCAGTATTCG |  |  |
|  | *aaiC* | aaiC-F | | ATTGTCCTCAGGCATTTCAC | 215 |  |
|  |  | aaiC-R | | ACGACACCCCTGATAAACAA |  |  |
|  | *aat* | _p_cvd432-F | | CTGGCGAAAGACTGTATCAT | 650 |  |
|  |  | _p_cvd432-R | | CAATGTATAGAAATCCGCTGTT |  |  |
|  | *iaa* | ial upper | | CTGGATGGTATGGTGAGG | 320 |  |
|  |  | ial lower | | GGAGGCCAACAATTATTTCC |  |  |
|  | *ipaH* | Shig-1 | | TGGAAAAACTCAGTGCCTCT | 424 |  |
|  |  | Shig-2 | | CCAGTCCGTAAATTCATTCT |  |  |
|  | *stx1* | stx1F | | CACAATCAGGCGTCGCCAGCGCACTTGCT | 606 | [3] |
|  |  | stx1R | | TGTTGCAGGGATCAGTGGTACGGGGATGC |  |  |
|  | *stx2* | stx2F | | CCACATCGGTGTCTGTTATTAACCACACC | 372 |  |
|  |  | stx2R | | GCAGAACTGCTCTGGATGCATCTCTGGTC |  |  |
| **ExPEC primers** | *focG* | focG_106F | | CGTACCTGTACCATTGGTAATGGAGG | 366 | [3] |
|  |  | focG_471R | | TGAATTAATACTTCCCGCACCAGC |  |  |
|  | *kpsMII* | kpsMII_121F | | GCGCATTTGCTGATACTGTTG | 452 |  |
|  |  | kpsMII_572 | | GGGAACATGATGCAGGAGATG |  |  |
|  | *papA* | papA_67F | | ATGGCAGTGGTGTCTTTTGGTG | 717 |  |
|  |  | papA_+202R | | CGTCCCACCATACGTGCTCTTC |  |  |
|  | *sfaS* | sfaS_210F | | GTCTCTCACCGGATGCCAGAATAT | 138 |  |
|  |  | sfaS_347R | | GCATTACTTCCATCCCTGTCCTG |  |  |
|  | *afa* | afa F | | GGCAGAGGGCCGGCAACAGGC | 594 |  |
|  |  | afa R | | CCCGTAACGCGCCAGCATCTC |  |  |
|  | *hlyD* | hlyD_92F | | CTCCGGTACGTGAAAAGGAC | 904 |  |
|  |  | hlyD_995R | | GCCCTGATTACTGAAGCCTG |  |  |
|  | *iutA* | iutA_674F | | ATCGGCTGGACATCATGGGAAC | 314 |  |
|  |  | iutA_987R | | CGCATTTACCGTCGGGAACGG |  |  |
| **ERIC Primer** | ERIC | ERIC-2 | | AAGTAAGTGACTGGGGTGAGCG | N/A | [4] |
| **Genes** | | | **Pathotypes** | | | |
| *estA* | | | ETEC | | | |
| *eltB* | | |  |  |  |  |
| *bfpA* | | | EPEC | | | |
| *eae* | | |  |  |  |  |
| *aaiC* | | | EAEC | | | |
| *aat* | | |  |  |  |  |
| *iaa* | | | EIEC | | | |
| *ipaH* | | |  |  |  |  |
| *stx1* | | | EHEC | | | |
| *stx2* | | |  |  |  |  |

**References:**

[1] T. Tabassum, M.S. Hossain, A. Ercumen, J. Benjamin-Chung, M.F. Abedin, M. Rahman, F. Jahan, M. Haque, Z.H. Mahmud, Isolation and characterization of cefotaxime resistant Escherichia coli from household floors in rural Bangladesh, Heliyon. (2024).

[2] Z.H. Mahmud, M.H. Kabir, S. Ali, M. Moniruzzaman, K.M. Imran, T.N. Nafiz, M. Islam, A. Hussain, S.A.I. Hakim, M. Worth, Extended-Spectrum Beta-Lactamase-Producing Escherichia coli in Drinking Water Samples From a Forcibly Displaced, Densely Populated Community Setting in Bangladesh, Front. Public Heal. 8 (2020) 228.

[3] M.S. Hossain, S. Ali, M. Hossain, S.Z. Uddin, M. Moniruzzaman, M.R. Islam, A.M. Shohael, M.S. Islam, T.H. Ananya, M.M. Rahman, M.A. Rahman, M. Worth, D. Mondal, Z.H. Mahmud, ESBL Producing Escherichia coli in Faecal Sludge Treatment Plants: An Invisible Threat to Public Health in Rohingya Camps, Cox’s Bazar, Bangladesh, Front. Public Heal. 9 (2021). https://doi.org/10.3389/fpubh.2021.783019.

[4] Z.H. Mahmud, S.Z. Uddin, M. Moniruzzaman, S. Ali, M. Hossain, M.T. Islam, D.T.D. Costa, M.R. Islam, M.S. Islam, M.Z. Hassan, Healthcare Facilities as Potential Reservoirs of Antimicrobial Resistant Klebsiella pneumoniae: An Emerging Concern to Public Health in Bangladesh, Pharmaceuticals. 15 (2022) 1116.
